# External Scaffold for Strengthening the Pulmonary Autograft in the Ross Procedure

**DOI:** 10.3390/biomimetics9110674

**Published:** 2024-11-05

**Authors:** Francesco Nappi, Aubin Nassif, Thibaut Schoell

**Affiliations:** Department of Cardiac Surgery, Centre Cardiologique du Nord, 93200 Saint-Denis, France; aubinnassif@gmail.com (A.N.); tiboschoell@hotmail.com (T.S.)

**Keywords:** ross operation, external support, bioresorbable reinforcement, polyethylene terephthalate, expanded polytetrafluoroethylene, extracellular matrix

## Abstract

Despite offering several potential benefits over standard prosthetic aortic valve replacement, the use of the pulmonary autograft has been limited to date due to concerns over the risk of pulmonary autograft expansion and the need for reintervention. Several techniques using materials with biomimetic potential have been developed to reduce this complication. The incidence, risk factors, and pathophysiology of pulmonary autograft dilatation are discussed in this article. This seminar will provide an overview of the techniques of external pulmonary autograft support and their advantages and limitations. It also considers future directions for further investigation and future clinical applications of external pulmonary autograft support. Dilatation of the autograft is more likely to occur in patients with aortic regurgitation and a dilated aortic annulus. External scaffolding may prevent autograft stretching and expansion in these specific cases. However, from a biomimetic point of view, any permanent scaffold potentially restricts the movement of the autograft root. This reduces some of the benefits associated with the use of autologous tissue, which is the priority of the Ross procedure. To address this issue, several bioresorbable matrices could be used to support the root during its initial adaptive phase. Control of blood pressure with aggressive therapy is the first line to avoid this problem in the first year after pulmonary autograft implantation, together with support of the annular and sinotubular junction in some selected cases. This is the best way to maintain stable autograft root dimensions while preserving root dynamics. However, to determine the efficacy of this combined external support and best medical management, it is important to perform regular imaging and clinical follow-up.

## 1. Introduction

In the event that an aortic valve (AV) replacement (AVR) is required, there are a number of potential replacement options. These may include the use of bioprosthetic valves, mechanical valves, an aortic valve homograft, or a pulmonary autograft as part of the Ross procedure. The selection of an aortic valve substitute has significant implications for long-term outcomes; therefore, it is crucial to exercise precision in determining the most suitable option to align with the individual patient’s specific necessities [1,2,3,4]. In countries with a high income per capita, the majority of patients who receive an AVR are elderly. In such cases, the selection of a suitable valve substitute is often a relatively headed process, as there is a substantial body of evidence demonstrating that standard surgical aortic valve replacement (SAVR) or catheter-based bioprosthetic valves using transcatheter valve implantation (TAVI) are associated with excellent short- and long-term results in this specific age group [3,4,5]. In stark contrast, the clinical profile of young and middle-aged adults diagnosed with AV disease represents a formidable clinical challenge. As a consequence of their greater longevity, these patients are subjected to a greater cumulative lifetime risk of valve-related complications, which is a result of their longer lifespan, allowing a greater opportunity for valve complications to arise [6].

In addition to the other factors that should be taken into account when selecting an aortic valve substitute, the level of physical activity that the patient will be undertaking must be considered. Furthermore, it should be noted that many young and middle-aged adult patients, in contrast to older patients, wish to pursue higher levels of physical activity after their operation [3,4]. Another fundamental point of consideration is the necessity for a maximum restoration of normal survival and the minimization of the risk of valve-related complications. Consequently, the optimal AV substitute for these young patients should also provide durable hemodynamic properties that permit an active lifestyle with an excellent quality of life [7,8,9,10].

Accordingly, the selection of an optimal diseased aortic valve replacement is predicated on the biomimetic capacity of the tissue utilized. The most frequently implanted valves in young and middle-aged adults, which are mechanical prostheses, lack biomimetic behavior despite being primarily utilized due to their ease of implantation and durability [11,12,13,14]. Moreover, conventional mechanical valves have been demonstrated to be thrombogenic, necessitating continuous anticoagulation throughout the patient’s lifetime. This presents a persistent risk of thromboembolic and hemorrhagic complications [15]. The management of antithrombotic therapy in women of childbearing age contemplating pregnancy who are fitted with a mechanical valve represents a further challenging aspect of clinical practice. In such cases, the potential benefit of biomimetic tissue may be particularly advantageous [16,17]. Despite the use of conventional stented bioprosthetic and aortic valve homografts providing a means of obviating the need for lifelong anticoagulation, these alternatives are devoid of biomimetic capabilities. This is due to their failure to exhibit a tissue remodeling process and biomechanics that are paramount characteristics of autologous tissue [18]. In consequence, these constitute “passive for biomimetic function” substitutes for AVR in patients presenting with severe aortic valve disease who are candidates for surgery. Additionally, when implanted in young adults, these biological substitutes are linked with a predictable higher incidence of structural valve deterioration, resulting in the need for repeated surgical intervention [19,20,21,22,23,24,25,26,27,28,29,30,31].

The use of autologous tissue with remodeling potential for aortic valve and root replacement has not been widely adopted, largely due to the dearth of comprehensive basic research. The industry has allocated the majority of its financial resources to the development of conventional mechanical and stented valves [32]. In light of these findings, several studies, both randomized and observational, have demonstrated superior outcomes in young to middle-aged adults when mechanical valves are utilized in comparison to bioprosthetic valves [19,21,33]. However, there has been a notable surge in the utilization of bioprosthetic valves for AVR in this age group over the past two decades [11,13].

Against this background, there has been a resurgence of interest within the cardiovascular community with regard to the potential of biomimetic tissue for facilitating remodeling. With this in mind, the utilization of pulmonary allografts in the Ross procedure represents a pivotal point of consideration [1,6,34,35]. Indeed, in recent years, a considerable body of research has demonstrated the effectiveness of the Ross procedure over the long term, as evidenced by a number of published studies [20,36,37,38,39,40,41,42,43,44,45,46,47,48,49,50,51,52,53,54,55,56], as regard both historical [45,46,47] and modern series [20,36,37,38,39,40,41,42,43,44,49,50,51,52,53,54,55,56]. As a result, there has been a resurgence of enthusiasm for this surgical technique. Given this renewed interest in the use of the Ross procedure, understanding the remodeling mechanism involving pulmonary autograft is of paramount importance.

In this review, we focus on various aspects of the biomodeling behavior of pulmonary autograft and review the current evidence supporting its use in selected young and middle-aged adults with aortic valve disease. We examined the structure and function of the external support in limiting pulmonary autograft expansion in the Ross procedure, as PA enlargement is a complication after implantation.

### Search Strategy

**Figure 1:**
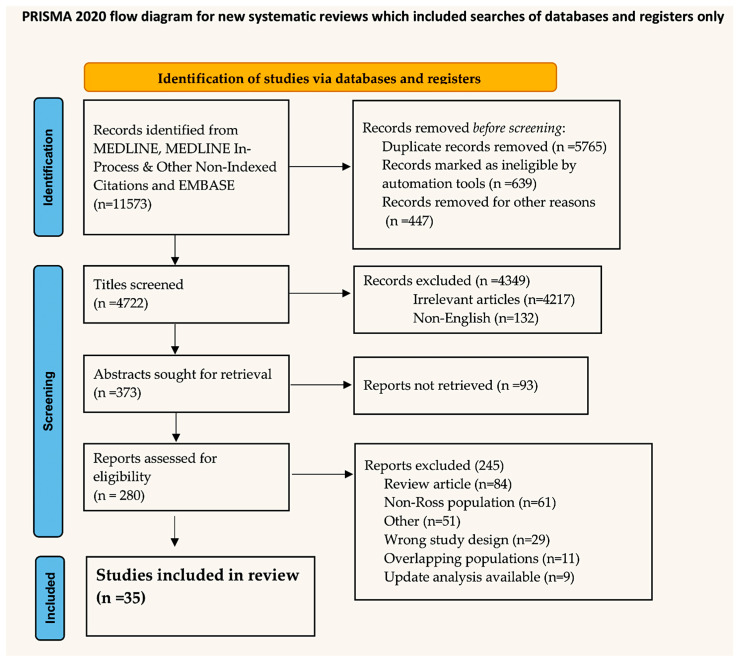
Flowchart.

In September 2024, PubMed, Ovid’s version of MEDLINE, and EMBASE scrutinized the systematic review using the terms “Ross procedure (25.613 to the present)” and coupled with extracellular matrix “(74 to the present)”, “circulating miRNAs (6 to the present)”, “external support (232 to the present)”, “reinforcement (180 to the present)”, “reinforced (180 to the present)”, and “living tissue (64 to the present)”. The search was able to isolate data from randomized controlled trials (RCTs), meta-analyses, observational studies, and articles from basic research. The review was registered with the OSF register of systematic reviews and followed the Preferred Reporting Items for Systematic Reviews and Meta-analyses (PRISMA) reporting guidelines. The project files are available online at https://osf.io/ezdy8/ (29 October 2024). A preliminary screening identified 11,573 manuscripts that were subsequently included in the PRISMA flowchart. The objective was to ascertain the total number of papers that would be subjected to the final evaluation. Prisma is reported in Figure 1.

## 2. The Historical Course of Pulmonary Autograft

The initial approach to utilizing a pulmonary autograft for the replacement of a diseased aortic valve while simultaneously implanting a homograft in the pulmonary position was first documented in human subjects by Donald Ross in 1967. Accordingly, the utilization of a PA implanted in the aortic position for the treatment of valve and left ventricular outflow tract pathology is designated as the Ross operation [57]. The preceding methodology, which was based on experimental research, has its origins in a groundbreaking paper published by Lower et al. [58]. Neither Ross and colleagues nor Lower and colleagues were cognizant of the possibility of utilizing implanted pulmonary tissue with remodeling potential in a biomimetic manner, particularly in the context of limiting its use to the pulmonic valve and pulmonary artery during surgical intervention. The late clinical results from the pivotal cohort of patients who underwent aortic placement of the pulmonary autograft to treat their diseased aortic valve clearly demonstrated the potentiality of remodeling of the autologous tissue. This process facilitates its biomimetic properties, effectively creating a living neoaortic root. It is notable that despite being implanted in a high-pressure environment, the pulmonary autologous tissue demonstrated prolonged structural integrity over time, with no evidence of early degeneration [45,46,47,48].

Following these initial results, a subsequent study of Car White and colleagues [59] demonstrated that, in contrast to the findings in PA, patients were at risk for homograft dysfunction when used to replace the pulmonary autograft, with the aim of reconstructing the right ventricle outflow tract. This manifested primarily as progressive valvular or supravalvular pulmonary stenosis, which was most frequently observed at the distal anastomosis and appeared to be an inflammatory process [59]. This phenomenon is associated with preoperative pulmonary hypertension, particularly when it is severe and/or irreversible, and represents a risk indicator for premature homograft degeneration. Conversely, patients presenting with mild pulmonary hypertension may be at reduced risk of autograft dilatation as a result of the pulmonary root undergoing a form of ‘pre-conditioning’. Pulmonary homograft stenosis follows a bimodal pattern, with an initial high-risk period lasting between 12 and 18 months. This is followed by a subsequent period of low-level but constant risk, extending over an extended timeframe [59].

In light of these considerable outcomes, the prevalence of PA utilization in Ross operations reached its zenith in the early 1990s, subsequently exhibiting a protracted decline over the subsequent two decades. Consequently, in addition to the failure of autografts, the biomimetic properties have been inadequately addressed, thereby underscoring the two primary factors that have contributed to this decline in popularity. The initial concern was the elevated complexity of the procedure, which elevated the operative risk in low-volume centers [60]. Additionally, there was a potential for long-term dysfunction of two valves [61], which would have placed individuals at risk for more invasive corrective procedures [62].

Notwithstanding the considerations previously outlined, a considerable number of surgical centers around the globe continued to perform the operation while conducting a comprehensive analysis of the long-term outcomes. The aforementioned results yielded a more sophisticated comprehension of pulmonary autograft remodeling and adaptation, along with an insight into biomimetic behavior in the context of systemic conditions. Additionally, they illuminated the underlying processes associated with pulmonary autograft and homograft failure. These combined efforts yielded iterative enhancements and adaptations to the surgical technique. These were subsequently reflected in the outstanding durability outcomes observed in more recent reports from high-volume, experienced centers. In light of the mounting evidence pointing to the favorable long-term outcomes of the Ross procedure, particularly in contrast to the less than optimal results associated with conventional AVR in the younger and middle age groups, there has been a resurgence in interest in the operation [20,36,37,38,39,40,41,42,43,44,49,50,51,52,53,54,55,56].

## 3. The Remodeling Properties of Pulmonary Autograft and Its Biomimetic Function

The utilization of pulmonary autografts to replace the aortic valve endows the implanted autologous tissue with biomimetic functionality. This approach is predicated on fundamental observations pertaining to the intricate structure and function of the aortic root. The underlying assumption is that due to the ability of autologous tissue to remodel, its utilization represents an effective treatment approach for aortic valve disease. The objective of this strategy is to develop an analogous living structure that maintains the mechanical and functional characteristics of the neoaortic root, with the ultimate goal of improving long-term clinical outcomes in individuals with disease.

It is established that the mechanics, as well as the functional behavior, of the aortic roots are intricate and complex. These are regulated by the synergic action of four main elements: the aortic annulus, the aortic leaflets, the sinuses of Valsalva, and the sinotubular junction. The structural properties of the individual elements and their overall integrity serve to define the remodeling potential of the PA as a biomimetic tissue.

### 3.1. Structural Building of the Aortic Root: General Concepts

Extracellular matrices (ECMs) are three-dimensional networks of macromolecules that are organized into a well-ordered and complex architectural structure. These structures play a pivotal role in regulating tissue organization and remodeling, in addition to influencing various cellular activities [63,64]. The fundamental components of these highly complex structures are collagens, proteoglycans (PGs), glycosaminoglycans (GAGs), elastin and elastic fibers, laminins, fibronectin, and other proteins and glycoproteins, including matricellular proteins [65,66]. ECMs serve as communication intermediaries between cells within organs and tissues, orchestrating the coordination of multiple signaling pathways to facilitate the transmission of both inside-out and outside-in commands [63,64]. Consequently, ECMs play an instrumental role in orchestrating tissue morphogenesis, development, and homeostasis. This is achieved by modulating fundamental cellular processes, including physiology, growth, survival, differentiation, and adhesion. Extensive remodeling of ECMs occurs in pathological states, wherein these macromolecules serve as pivotal drivers of disease exacerbation and progression [67,68,69,70]. The formation of ECM components can be continuously modified according to biochemical and/or mechanical stimuli, thereby enabling a precisely regulated, dynamic process of ECM remodeling [71].

The human body contains a multitude of elastic fibers, which are found in various organs, including the arteries, lungs, and skin. In the context of the circulatory system, the elastic property is of paramount importance for the maintenance of a steady blood flow and pressure generated by the heart. Tropoelastin (TE), which is released from cells that are capable of producing elastin, forms the elastin meshwork through cross-linking on a scaffold of fibrillins and other microfibril proteins [72]. Additionally, fibrillins facilitate the binding of proteins to elastin, yet they also engage in cell signaling through their interplay with syndecans and integrins and the storage of the transforming growth factor β (TGF-β) family of growth factors in the matrix [73]. The formation of elastin occurs during the developmental and juvenile stages of life, and its decomposition commences during the adult and age-related decline phases [74]. The proteolytic action of elastases on elastin-derived peptides (EDPs) has been demonstrated to influence signal transduction, thereby affecting the physiological maintenance of arteries [75].

The initiation of covalent cross-linking between TE and collagen fibrils is catalyzed by lysyl oxidase (LOX) and LOX-like (LOXL) proteins, which in turn stabilize the associated networks (see reference [76]. Furthermore, they can function as signaling mediators due to their interaction with various growth factors (GFs), including fibroblast growth factor 2 (FGF2) and transforming growth factor beta (TGF-beta), or due to the oxidation of platelet-derived GF beta (PDGF beta). LOX and LOXL are involved in a number of biological processes, including development, tissue repair, and remodeling [76]. Their expression profiles are influenced by a range of pathological factors. The regulation of these proteins can be influenced by ECM proteins, inhibitors, and PGs. As an illustration, studies have demonstrated that fibromodulin and syndecan-4 can enhance the interaction of LOX proteins with collagens [77]. The recent study has identified thrombospondin-2 as a modulator of skin elasticity and has also proposed that it functions in the pulmonary artery remodeling process [78]. The knockdown of thrombospondin-2 resulted in a reduction in both collagen fibrillogenesis and levels of LOX [79].

Another macromolecule capable of forming supramolecular assemblies is fibronectin, which plays a role in regulating mechanical properties, including those related to tension. This is accomplished through conformational changes in its fibers, which can be classified as active-stretching or relaxed fibronectin fibers. Additionally, fibronectin engages in interactions with various cellular receptors and ECM molecules, including integrins such as EphrinB2, which regulate cellular adhesion. It also interacts with growth factors (GFs) and cytokines, which play an important role in cell growth, differentiation, and migration [80,81].

The laminin family comprises over 16 members, with each molecule comprising three chains: α, β, and γ. Distribution patterns of these laminins exhibit tissue- and cell-specific variations. Notably, laminin-111 is predominantly expressed in embryos, whereas laminins 521 and 511 are more prevalent in adult tissues. Laminins 211 and 221 exhibit a more restricted localization, being detected in the basement membrane (BM) of cardiac muscles, whereas laminins 411 and 421 are identified in the basement membrane of endothelial cells [82,83].

**Figure 2:**
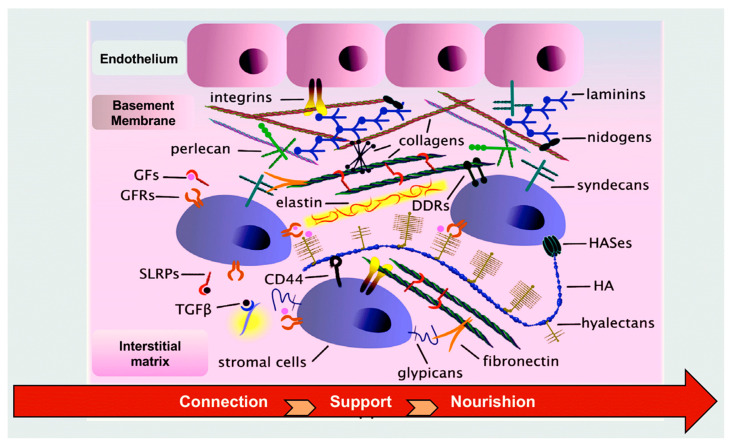
Composition and function of the extracellular matrix [63,64,65,66,67,68,69,70,71,72,73,74,75,76,77,78,79,80,81,82,83,84].

Members of the tenascin (TN) family belong to the group of matricellular proteins, which comprise four distinct members: TN-C, TN-R, TN-W, and TN-X. TNs encompass three distinct domains: EGF-like, fibronectin-type III, and a fibrinogen-like globular domain. A number of these functional domains engage in interactions with a variety of other extracellular matrix (ECM) proteins, including collagens, fibronectin, fibrillins, proteoglycans (PGs), growth factors (GFs), chemokines, and other soluble factors. Additionally, TNs regulate cellular adhesion via their interaction with integrins. These proteins are involved in a number of important biological processes, including embryonic development and pathogenesis, as well as tissue homeostasis [84] (Figure 2).

### 3.2. Living Aortic Root

The aortic valve is traditionally regarded as a fixed component that moves in response to transvalvular pressure gradients. Clinical and experimental evidence, however, has demonstrated that the whole structure of the aortic root is a dynamic, living frame that interacts to form a self-sustaining, functional assembly. The pulmonary autograft is the sole available substitute capable of ensuring the long-term viability of the neoaortic valve. In contrast, all other aortic valve substitutes are composed of non-living tissue. Even homovital homografts, which are unprocessed homografts obtained under sterile conditions, stored in tissue-culture medium, and inserted at the earliest possible convenience, were once considered to preserve long-term viability. Nevertheless, they have demonstrated to lack cells within a span of a few weeks following implantation [20,59].

A considerable body of research has explored the developmental disorders of ECM components and aortic root mechanics by integrating genetic mechanisms and the phenomenon of mechanotransduction [85,86,87,88,89,90,91,92]. It is of the utmost importance to gain insight into the interrelationship between these events in order to enhance one’s comprehension of developmental disorders and aortic root mechanics. The objective of these studies has been to elucidate the role of each component in the systolic–diastolic cycle of the heart, with a particular focus on its functional and genetic aspects.

In a study conducted by Lu et al. [88], the deletion of the gene *Sox17* in the aortic root endothelium of mice was observed to result in an underdeveloped aortic root, which subsequently led to the formation of a bicuspid aortic valve. The absence of the non-coronary leaflet and the mispositioned left coronary ostium are the underlying causes of this phenomenon. The deletion of *Sox17* has the effect of inhibiting the transcription of Pdgfb by the endothelium and the growth signaling of PDGFB to the mesenchymal cells of the non-coronary leaflet. The restoration of PDGFB in the aortic root endothelium has been observed to rectify the defects in the non-coronary leaflet and left coronary ostium in mice lacking *Sox17*. These findings suggest that a relationship between SOX17 and PDGFB may be involved in aortic root development, which is crucial for the patterning of the aortic valve and coronary ostium. This may provide insight into a potential shared disease mechanism for concurrent anomalous aortic valves and coronary arteries.

A landmark study conducted by Dagum and colleagues [89] provided experimental evidence of the complex three-dimensional distortions of the aortic root throughout the cardiac lifecycle. The aforementioned strains and contractions exert a favorable effect on the annulus, sinuses of Valsalva, and sinotubular junction, thereby reducing aortic cusp stress and enhancing shear flow during systole while also increasing coronary flow reserve during both systole and diastole. The results led to improvements in several areas related to valve replacement. Techniques for repairing or replacing the valve using unstented allografts or xenograft tissue valves that preserve the normal dynamics of the aortic root have the potential to result in a reduced long-term risk of cusp deterioration. Similarly, El Hamamsey et al. [90] conducted a detailed analysis of the microstructure of aortic valve leaflets, emphasizing the intricate and sophisticated nature of the aortic root. The leaflets are populated by a monolayer of valvular ECs on both the ventricular and aortic sides. The body of the leaflet comprises a mixed population of valvular interstitial cells, including smooth muscle cells, fibroblasts, and myofibroblasts, which collectively constitute the extracellular matrix. By means of a process designated as mechanotransduction, which transforms mechanical stimuli into biologic signals, endothelial cells of the valve are capable of sensing and responding to alterations in shear stress. Additionally, signaling from the endothelium can modify the mechanical properties of the aortic valve leaflets in response to their humoral environmental context [89]. Furthermore, valvular interstitial cells are responsible for the generation, maintenance, and repair of the extracellular matrix, which is composed primarily of elastin, collagen, and glycosaminoglycans and exhibits both secretory and contractile properties [91]. At last, a complex network of intrinsic nerves has been identified by microscopic examination, which is thought to play a significant role in regulating the aortic valve’s responses to various hemodynamic and humoral stimuli [92].

It has recently been demonstrated that multiplexins play a significant role in the development of cardiac structures, such as the left ventricular outflow tract. This collagen subfamily includes two members, collagens XV [93] and XVIII [94,95], which both consist of multiple triple-helical domains embedded within a framework of noncollagenous domains and are characterized by the presence of GAG chains. Collagen XV is responsible for linking striated collagen fibers situated beneath the BM and plays a role in regulating cell interactions [96], such as adhesion and migration [97]. A deficiency in collagen XV has been linked to the development of cardiomyopathy [98], while the absence of collagen XV has been observed to confer protection against ischemic stroke in mice [99]. Collagen XVIII is essential for maintaining basement membrane integrity. It regulates cell survival, stem or progenitor cell maintenance and differentiation, and inflammation during the remodeling process of the ECM [95].

## 4. Adaptive Pulmonary Autograft Remodeling

The dynamic nature of the extracellular matrix (ECM) is essential for the proper functioning of tissues, including their development, remodeling, repair, regeneration, and maintenance of homeostatic balance [67,71]. Any disruption to this intricate equilibrium has the potential to result in either adaptive or pathological processes [70,100,101]. Following implantation in the aortic position, the pulmonary autograft exhibits adaptive remodeling as a result of its viability, thereby mimicking the intricate anatomy and functionality of the native aortic root. The process can be facilitated by systemic pressure or specific biochemical reactions [63,102,103,104,105,106,107,108,109,110]. The remodeling of pulmonary arteries is a complex phenomenon that involves a synergistic action mediated by elastic fibers and elastin, laminin, and integrin. Additionally, critical enzymes involved in remodeling contribute to the dynamic character of the extracellular matrix (ECM).

### 4.1. The Remodeling of ECM-Based Tissue Elasticity: The Role of Elastic Fibers and Elastin

Elastic fibers are the extracellular assemblies responsible for the necessary elasticity and extensibility. They are indispensable for the physiological functionality of a multitude of organs, including the arteries, dermal tissue, tendons, and pulmonary structures, which undergo reversible and repeated deformation. Elastic fibers comprise two morphologically discernible elements: a mantle of longitudinally aligned fibrillin-based microfibrils and a dense core of cross-linked elastin, which constitutes over 90% of the fiber content. The microfibrils are filamentous structures, measuring 10–12 nm in width, which exhibit a distinctive “beads-on-a-string” morphology [111]. The aforementioned structures provide tissues with long-range elasticity, a quality that is particularly enhanced when elastin is deposited on a microfibrillar scaffold. Microfibrils are primarily composed of fibrillins [112], although several other proteins have been identified as being associated with them [111]. The aforementioned categories of molecules include microfibril-associated glycoproteins (MAGPs), [113] elastin microfibril interfaces, [114] a disintegrin and metalloproteinase with thrombospondin motifs (ADAMTS) and ADAMTS-like proteins [115], as well as latent transforming growth factor-β binding proteins (LTBPs, including LTBP-4) [116].

The other significant component is elastin, which is an insoluble biopolymer composed of units derived from its soluble precursor, TE. TE’s primary structure is distinguished by alternating hydrophobic and hydrophilic domains, which are encoded by discrete exons. Consequently, TE’s domain structure reflects the exon organization of the gene. It has been observed that hydrophilic domains possess either lysyl-alanine (KA) or lysyl-proline (KP) motifs [117]. These domains are known to be involved in the covalent cross-linking process, which is induced by LOX or LOXL enzymes. This ultimately leads to the formation of mature elastin. In contrast, the hydrophobic domains are responsible for elasticity and are involved in cell interactions [118]. The primary transcript of elastin is subject to extensive alternative splicing, giving rise to a multitude of isoforms that do not affect the reading frame. The expression of numerous isoforms in human tissues has been demonstrated in a number of reports [119].

In healthy conditions, such as during the remodeling of pulmonary autografts that has occurred in response to adaptation, mature elastin is metabolically stable over the lifespan of the species. Its half-life in humans has been quantified as exceeding 70 years [120]. One of the factors contributing to this remarkable durability is elastin’s notable resilience to proteolysis, which is primarily attributed to its extensive cross-linking and the remarkable density of molecular packing. In its fully mature state, elastin assumes a hydrophobic character and becomes completely insoluble. However, its hydrophobic hydration is indispensable for the manifestation of its elastic properties [121].

The process of elastogenesis begins with the transcription and translation of fibrillins, which then assemble into microfibrils. These microfibrils serve as a scaffold for the subsequent TE deposition [122]. It has been observed that fibrillin networks are capable of undergoing a process of cross-linking. This results in the stabilization of the three-dimensional bundle structure. The cross-links that have thus far been reported are inter-molecular disulfide bonds [123] and e(c-glutamyl) lysine cross-links. The latter are catalyzed by members of the transglutaminase family [124].

After the remodeling process of PA, the ramifications of elastin’s molecular aging are manifold, encompassing both direct repercussions on the structural and mechanical attributes of this matrix protein and indirect consequences on cellular processes [125,126,127].

**Figure 3:**
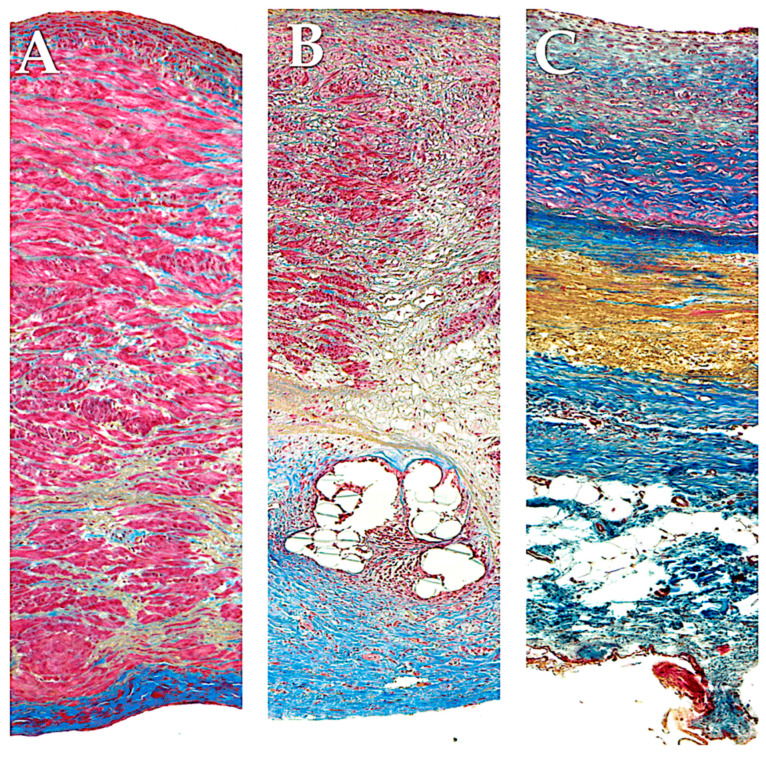
Full-thickness wall reconstruction of unreinforced PA (**A**), PA externally reinforced with non-resorbable polyethylene terephthalate (**B**), and PA reinforced with externally resorbable polydioxanone (**C**). Masson’s trichrome staining. Red, smooth muscle cells; blue, collagen fibers; yellow, elastic fibers. Histological analysis of pulmonary autografts with and without remodeling revealed significant differences between non-resorbable and resorbable reinforced PA. (**A**) The higher systemic pressure determines the intimal denudation and media disruption in the PA without external reinforcement. Smooth muscle cells were visible in the media. These cells had irregular profiles and no discernible alignment and were widely spaced with intervening collagen fibers grouped in thick and dense bundles. Deeper in the media, sparse elastic fibers formed irregular fascicles, and the adventitia was composed of dense connective tissue. (**B**) The external polyethylene terephthalate promoted the development of a foreign body inflammatory reaction around the material, and the phenomena of transmural and endoluminal migration of the mesh cutting through the PA wall were noted. On histology, a prominent inflammatory infiltrate was seen, and fewer smooth muscle cells with more interstitial connective tissue could be seen in comparison to the control group. (**C**) External reinforcement with resorbable polydioxanone showed no signs of an inflammatory reaction. Reorganization of the media was evident with preservation of the endothelial lining. Immediately beneath the intima, smooth muscle cells were seen intertwined with collagen fibers, whereas deeper collagen bundles intertwined with elastic fibers, forming a thick, highly organized layer of concentric lamellae. Loose connective tissue with adipocytes formed the tunica adventitia [86,87]. Adapted with permission Order Number 501943363.

In addition to chemical modifications, elastin is subject to mechanical fatigue processes [128,129]. The damage or even rupture of elastin causes a reduction in the function of elastic fibers and results in the transfer of mechanical stress to other extracellular components, such as collagen fibers, which has a significant impact on tissue mechanics [130]. Furthermore, the deterioration of elastic fibers is exacerbated by the activity of various extracellular proteases, including elastases. These proteases belong to three classes of families: serine proteinases with cathepsin G, proteinase 3, and neutrophil elastase; [131,132,133] MMPs, including MMP-2, -7, -9, -12, and -14; [134,135,136] and the cysteine proteinases cathepsins K, L, S, and V [137,138]. In addition to the functional impairment, elastin degradation results in the secretion of bioactive peptides, designated “elastokines,” which belong to the matrikine family [139,140] (Figure 3).

### 4.2. Laminins in the Remodeling Process: The Fundamental Three-Armed ECM Adhesion Proteins

Laminins are high-molecular-weight (400–900 kDa) heterotrimeric adhesion proteins that are found in BM. Blood vessels are surrounded by a thin sheet of extracellular protein structures, known as BMs, which are highly specialized and surround not only the vessel wall but also muscle, fat, and Schwann cells. Essentially, laminins are critical for the formation and function of BMs by self-polymerizing into a cell-associated network. Laminins consist of one α, one β, and one γ-chain, each encoded by a different gene, and are found in worms, flies, and mammals. In vertebrates, five α, three β, and three γ chains have been identified, which may be combined into more than 16 different isoforms. Laminins are designated according to their chain configuration, and prototype laminin-111 (composed of an α1, β1, and γ1 chain) was the first laminin isoform discovered more than 40 years ago. Overall, laminin expression is tissue-specific, and BMs have at least one laminin isoform. The connection between the network-forming collagen type IV and laminin is well documented, particularly between non-fibrillar collagen and laminin 2 (α2β1γ1), laminin 4 (α2β2γ1), and laminin 5. The α2β2γ1 and laminin 10 (α5β1γ1) are distinctive to humans and are found in the blood vessels, cardiac, skeletal muscle, and smooth muscle tissues [141,142,143,144,145,146,147].

The structures of the integrin- and dystroglycan-binding fragments of laminins have been elucidated at the atomic level. Laminins are attached to the cell surface by binding the LG domains (α-chain C-terminal globular domain) to various cell surface receptors, such as integrins and α-dystroglycan, to name a few. Laminins interact with a variety of other BM proteins (e.g., perlecan, nidogen, and agrin). Thus, laminins constitute supramolecular networks essential to embryonic development and multiple adult organs and systems [141,148,149,150,151,152,153]. The tissue-specific deletion of laminin α5 has also demonstrated essential functions in the micro- and macrovascular endothelial function, lung, neuromuscular junction, and kidney. In conclusion, laminins are of paramount importance for the maintenance of BM integrity, early embryonic development, organogenesis, and the sustenance and survival of numerous tissues. Over the past decade, it has also become evident that recombinantly expressed laminins serve as crucial tools in the generation of xenogeneic-free as well as defined cell differentiation and remodeling protocols [154].

### 4.3. Integrins: Function as Mediators of Adhesion and Signaling Between ECM and Cells

#### 4.3.1. Structure of ECM Integrins and Ligands

Integrins constitute a superfamily of transmembrane cell adhesion proteins that facilitate the linkage between the ECM and the cytoskeleton of cells. Integrins play a pivotal role in the transduction of intracellular signaling pathways and in interactions with ECM molecules. These proteins assemble into heterodimers comprising α-subunits and β-subunits with at least twenty-four distinct combinations. Integrins are composed of α-subunits, which are divided into 18 distinct types, and β-subunits, which are further classified into eight groups [155]. Integrin α- and β-subunits are type I transmembrane glycoproteins (TMEMs) comprising a sizable extracellular domain, a single TM domain, and a brief cytoplasmic domain [155]. Among the different subunits, some are more prevalent in heterodimers, with α1 present in twelve distinct heterodimers and αv present in five [156]. It is the extracellular domain, in particular the αI domain, that confers ligand selectivity to a number of different ECM macromolecules or counter receptors that are located on adjacent cellular surfaces. The four main categories can be further subdivided as follows: (a) the arginine–glycine–aspartic acid (RGD) motif, (b) laminin receptors, (c) leukocyte-specific receptors, and (d) collagen receptors. Integrin heterodimers exhibit ligand specificity due to their ability to bind to ECM ligands in either both subunits or the α-specific domain of the α-subunit. Furthermore, in the case of hematopoietic cells, the distinct combinations of various α-subunits and β2 integrins contribute to the ligand specificity of these heterodimers. In the context of RGD-binding integrins, the RGD ligand interacts with an interface between the a- and b-subunits. The R residue engages with a cleft in a β-propeller module in the α-subunit, while the D coordinates a cation that is bound in a vWF A-domain in the β-subunit [157]. A further acidic motif, designated as LDV, is believed to be functionally associated with RGD. Despite the absence of structural data, there is considerable evidence that it may bind in an analogous manner to RGD at the interface between the α- and β-subunits. It has been demonstrated that fibronectin, VCAM-1, and MAdCAM-1, which contain the LDV motif, bind to α4β1, α4β7, and α9β1, as well as to β2 subfamily and αEβ7 integrins [5,16]. The insertion of an A-domain in the α-subunit confers ligand binding specificity to numerous β-subunit families, including the β1-, β2-, and β7-subunits. With regard to β2 family-specific ligand sites, they exhibit structural similarities to the LDV motif. The primary distinction between β1/β7 ligands is that β2 utilizes glutamate in lieu of aspartate for cation coordination [5,19]. The A-domain, derived from α1-, α2-, α10-, and α11-subunits, forms heterodimers with β1, thereby creating laminin- and collagen-binding families [158]. Specifically, the α 2 A domain interacts with the triple helix of collagen via a GFOGER motif [159]. Conversely, non-αA domain-containing integrins, including α3β1, α6β1, α7β1, and α6β4, demonstrate a high degree of selectivity for laminin ligands.

#### 4.3.2. Overview of Integrin Activation and Roles in Physiology and Disease

The initial observation of integrin regulatory processes occurred in blood cells [160]. Integrins in platelets and leukocytes have been most extensively studied, yet they are present in numerous other cell types, playing a pivotal role in processes such as angiogenesis, cell migration, and extracellular matrix remodeling. The process of integrin activation involves the binding of talin to the cytoplasmic tail of the β1-subunit [161]. The binding of talin results in a structural alteration of both subunits, whereby the cytoplasmic region is separated and the extracellular region is extended. This enables a higher affinity with the ligands. Following the preliminary attachment of talin to the cytoplasmic domain, additional regulatory proteins bind to the cytoplasmic domain, thereby facilitating the activation and clustering of the protein with various adhesive molecular complexes [162]. Kindlin plays a significant role in inside-out integrin signaling, influencing ECM interactions and cell spreading. Its involvement in these processes can be attributed to two main functions: the activation of the β-subunit cytoplasmic tail and the recruitment of focal adhesion molecules such as paxillin. These actions lead to the activation of the RHO GTPase RAC1 and the direct polymerization of actin by the Arp2/3 complex, resulting in cell spreading [163,164]. Talin-1 and -3, two tensin proteins, are responsible for maintaining talin-induced integrin activation during adhesion maturation through binding to the β1-subunit. While the precise mechanism of integrin–tensin binding is yet to be fully elucidated, there is a clear transition from talin binding to tensin binding as the process of adhesion maturation progresses. This is due to a significant degree of overlap between the binding sites of the two proteins on the β1-subunit, as previously demonstrated [165]. Integrins mediate cell–extracellular matrix interactions, which in turn result in the formation of complexes that regulate downstream signaling pathways. These include the activation of focal adhesion kinase (FAK), SRC, AKT, and extracellular signal-regulated kinases (ERK) pathways, as well as small GTPases of the RHO family [166,167]. The aforementioned cascades are of paramount importance for integrin-mediated cellular behaviors, including cell death or survival, regulation of cytoskeletal dynamics, cell migration via the control of cell polarity, and maintenance of tissue integrity [168].

### 4.4. Mechanism of Adaptive Pulmonary Autograft Remodeling

Following placement in the aortic position, the pulmonary autograft initiates a process of adaptive remodeling due to its viability, resulting in anatomical and functional characteristics analogous to those of the native aortic root. This process can be facilitated by systemic pressure or specific biochemical reactions [1,5,34,85,86,87,169].

A preliminary investigation posits that when the pulmonary infundibulum is repositioned to the aortic position—in lieu of its natural location—it demonstrates a remarkable 30% potential for distensibility in comparison to the typical aortic root. This allows for a significant degree of distortion without compromising valve functionality [18]. The remodeling process is principally initiated and sustained by valvular endothelial and interstitial cells. When encountered by systemic circulation, these cells become activated and exhibit phenotypic modifications. As an example, pulmonary autografts implanted in the aortic position begin to express EphrinB2, as observed in ECs. Eph receptors and their ephrin ligands play a pivotal role in vascular development, a process that is indicative of left-sided heart valve endothelium but not right-sided. The expression of EphrinB2 triggers remodeling of the extracellular matrix, leading to increased levels of smooth muscle actin [89]. This is one of multiple possible pathways by which pulmonary autograft leaflets, when placed in the aortic position, undergo reversible phenotypic alterations to adapt to the mechanical forces of their novel environment and adopt the attributes of normal aortic valve leaflets. As a result, the thickness and breaking strain of pulmonary autograft leaflets become more comparable to those of native aortic valve leaflets [170].

The proteomic characteristics have been examined to elucidate the mechanisms underlying the dilated pulmonary autograft tunica media in comparison to the normal pulmonary artery and aorta tissue [164]. It was observed that a number of proteins responsible for specific functions of the vessel wall were significantly upregulated in unreinforced and dilated pulmonary autografts. The genes responsible for proteins associated with focal adhesion, the cytoskeleton, and the regulation of metalloproteases and proteoglycans were found to be downregulated. Additionally, microfibril-associated glycoprotein 1, which plays a role in regulating elastic fiber accumulation, demonstrated a notable decline. It was observed that there were marked alterations in proteins involved in the regulation of cellular signaling. This study found that the soluble Jagged-1 fragment and ectodysplasin-2 receptor increased in abundance, while the Notch-1 intracellular domain fragment decreased. The PA demonstrated different levels of Paxillin, Vimentin, Jagged-1 fragment, and Notch1 intracellular domain fragment in comparison to the control aorta. It is therefore proposed that there has been maladaptive remodeling in an expanded, non-reinforced pulmonary autograft [164].

As previously reported in our experimental studies using polydioxanone bioresorbable scaffolds, we have demonstrated the capacity to enhance this remodeling process in PA. The interplay between bioresorbable reinforcement and PA enabled a sophisticated vascular remodeling process, orchestrated by a harmonious balance between inflammatory response and the production of ECM. The remodeled structure exhibited analogous traits to those observed in the aorta yet retained biological activity and the capacity for growth. The histological analysis of the ECM in the strengthened PA revealed an increase in elastin filament content and a more consolidated structure of collagen fibers in the elastic zone of the vasculature. It is noteworthy that the metalloprotease MMP-9 was overexpressed [85,86,87].

## 5. Outer Reinforcement Support of Pulmonary Autograft to Prevent Dilation: Biomimetics vs. Nonbiomimetic Constituents

The use of the PA has long been regarded as a potential cause of long-term dysfunction of the aortic and pulmonary valves, particularly in patients initially presenting with single-valve disease. This vulnerability stems from the fact that the PA must accommodate pressures that exceed the normal range for which it is designed.

### 5.1. Technical Consideration to Improve Remodeling and Biomechanics

**Figure 4:**
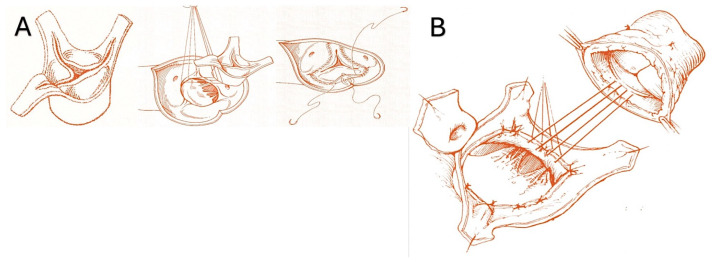
(**A**). Subcoronary technique. (**B**) Full root replacement technique [1,52].

**Figure 5:**
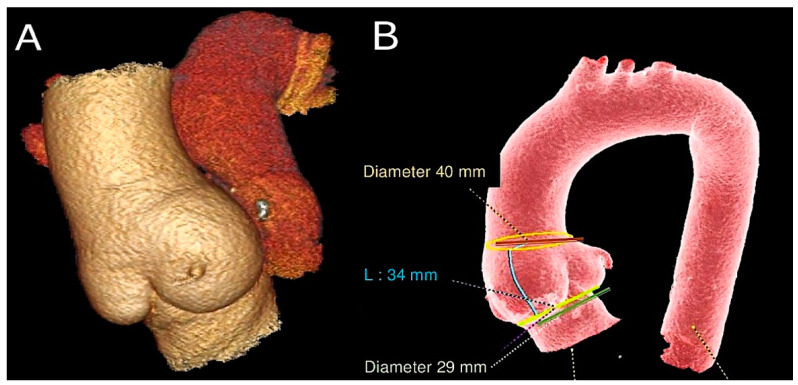
This CT scan shows (**A**) pulmonary autograft and pulmonary homograft from a Ross operation performed 23 years ago. (**B**) Measurements of PA were taken at the sinotubular junction, sinuses of Valsalva, and aortic annulus [51,52].

The pulmonary autograft can be implanted in one of two ways: the subcoronary implantation or free-end technique [38,46,47,48,57] and the root replacement procedure, also known as the miniroot or full root technique [20,50,51,52,54,169,171]. Furthermore, two additional techniques exist for harvesting the pulmonic valve for pulmonary autograft explantation. (Figure 4A,B, as well as Figure 5A,B).

The subcoronary technique entails the removal of the pulmonic valve and its subsequent implantation with only the leaflets and annulus (Figure 4A). In contrast, full root implantation involves the implantation of the pulmonary valve with the pulmonary root, thereby forming the neoartic root.

It is standard practice to reserve subcoronary implantations for individuals who have reached full physical development. In order to ensure success, long-term follow-up is essential [41]. The removal of surrounding muscle and connective tissue up to the annulus of the pulmonary valve has been shown to reduce the shear stress exerted by the transvalvular gradient [38,41,46,47,48,57]. It is notable that in the majority of centers worldwide, the subcoronary implantation technique has been superseded for several reasons, including the complexity of the implantation technique, less biomimetic behavior, and biomechanical concerns. Therefore, the most optimal option would be to implant in the root replacement position. This conclusion is derived from the aggregation of findings across a large series of patients [9,20,36,37,41,42,43,54,169,171,172].

The miniroot technique entails the transposition of the pulmonic valve and its pulmonary trunk into the aortic position, thereby effecting the removal of the pulmonary artery from the infundibulum of the right ventricle while ensuring that its morphology remains unaltered (Figure 4B). The pulmonary infundibulum is principally constituted by the conal or infundibular septum, which serves to separate the pulmonary valve from the aortic and tricuspid valves. The trabecula septomarginalis, also known as Leonardo da Vinci’s moderating beam, is composed of three distinct parts: the primary structure, an anterior extension or division, and a smaller, superior extension of the trabecular septum (Figure 5). The optimal mechanics of PA are contingent upon the integrity of the trabecula septomarginalis.

As previously hypothesized, the utilization of a pulmonary autograft as a miniroot implant is associated with a significant concern: an elevated risk of late pulmonary autograft dilatation due to the exposure of the entire root to elevated systemic pressures. This expansion, in turn, can lead to subsequent late autograft insufficiency at the site of unsupported pulmonary sinuses, the aortic annulus, and sinotubular dilatation. In their study, Horer et al. [173] observed a different rate of expansion of the PA root, which was statistically significant at the level of the neoaortic sinus (0.5 ± 0.1/year, *p* < 0.001) and the sinotubular junction (0.7 ± 0.2, *p* < 0.001), but not at the level of the annulus (0.1 ± 0.1, *p* = 0.59) with a mean follow-up period of 5.1 years.

### 5.2. External Support

**Figure 6:**
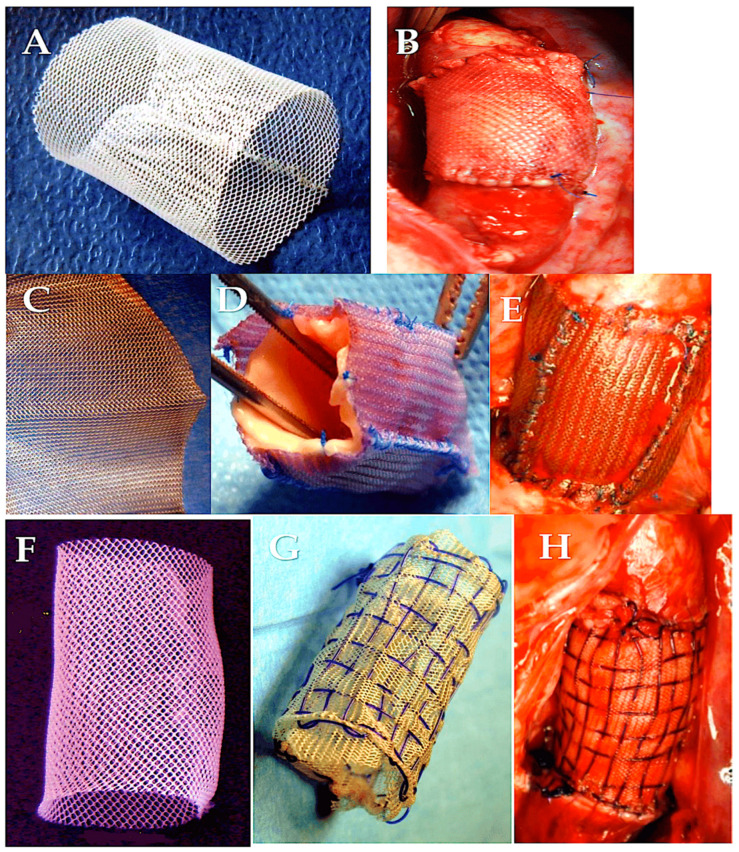
The PA can be strengthened with the help of external support (**A**–**H**). (**A**,**B**) Polyethylene terephthalate or poly(ethylene terephthalate), PET, and PETE. (**C**–**E**) Polydioxanone (PDS-ethicon). (**F**–**H**) Polydioxanone and polyglactin double-interlaced external reinforcement. Abbreviations: PA, pulmonary autograft [86,87]. Adapted with permission Order Number 501943363.

**Figure 7:**
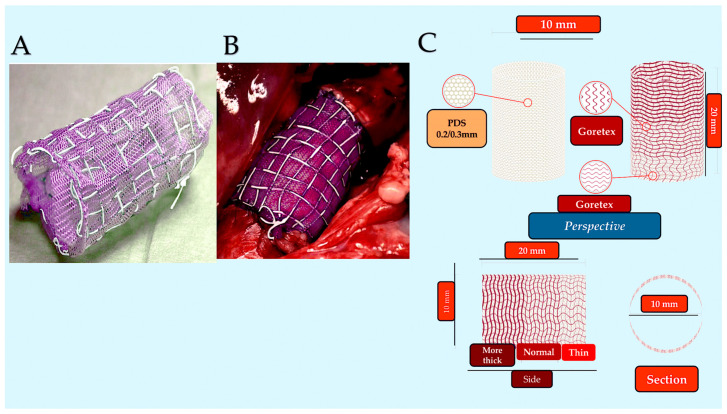
(**A**,**B**) Handmade PDS/PTFE (Gore-Tex) composite reinforcement (WL Gore & Associates, Newark, Del) prior to implantation. (**C**) Concept and design of a composite resorbable armored bioprosthesis. The specific design of Gore-Tex armor allows for multidirectional growth and resistance to dilatation. The unique weave of the upper armor gradually adapts and functionally compensates for autograft growth characteristics. Bottom red box on the left: initial implantation. Red box in the middle: intermediate phase. Red box on the right: full development. Note the progressive resorption of the resorbable layer and the progressive expansion of the unitary elements that make up the mesh, which is composed of the auxetic material ePTFE [86,87]. Adapted with permission Order Number 501943363.

In order to circumvent the potential hazard of pulmonary autograft malfunction and the necessity for further intervention, a number of technical modifications have been proposed. However, there is currently no consensus regarding the optimal methodology for their implementation. Higher-volume centers typically employ one of three distinct techniques, yet a paucity of data exists to substantiate the long-term efficacy of these methodologies. The different external supports used are shown in Figure 6 and Figure 7.

The incorporation of the pulmonary artery within the patient’s aortic root affords the autograft protection against the deleterious effects of systemic pressure over time [41,174]. Although reducing the size of the dilated aortic annulus may serve to mitigate early expansion, it does not prove an effective method of preventing late disfunction, which is likely attributable to pre-operative aortic failure. Recently, a reinforcement of the PA with an external Dacron graft (Polyethylene terephthalate or poly(ethylene terephthalate), PET, and PETE) has been proposed as a means of preventing late dilatation and failure. This objective is accomplished either by including the entire miniroot or by wrapping only the sinotubular junction without fully encompassing it [175]. Likewise, polyethylene terephthalate mesh (exostent) with a pore size of 0.7 mm is used in an experimental ovine model. The mesh was loosely placed around the lung autograft during surgery. The addition of an external exostent changes the mechanical behavior of the composite pulmonary autograft combined with the exostent [176]. It may therefore be able to bring the stresses in the pulmonary arterial wall closer to their homeostatic value, reducing the occurrence of growth and remodeling reactions. The elastin is compressed under the sheath, as was also observed [177]. Placement of the wrapping around the PA causes atrophy of the smooth muscle cells, resulting in more densely packed elastin. The thickness of the PA is reduced, but the addition of the sheath and the fibrotic reaction results in an overall thickness that has not changed dramatically.

The personalized external aortic root support (PEARS) system represents a novel surgical technology that employs three-dimensional printing techniques to create a patient-specific model of the aorta. The bespoke reproduction is derived from a magnetic resonance imaging (MRI) or computed tomography (CT) scan and is composed of a polyethylene terephthalate mesh. Initially conceived as a treatment for root aneurysms in patients with Marfan syndrome [178], the technique involves wrapping the graft around the native aortic root, thereby stabilizing it and limiting future growth [179]. This approach has yielded encouraging outcomes, demonstrating stability of aortic root dimensions at mid-term follow-up and the absence of acute aortic complications in patients with Marfan syndrome [178,180,181]. These promising findings suggest the potential for extending this technology to pulmonary autografts. Despite the lack of human trials, preclinical investigations have employed a sheep model to evaluate the potential for pulmonary artery translocation into the descending aorta. The initial outcomes validated the stabilizing effect of the exostent in comparison to unassisted pulmonary artery segments [176]. The histological examination of the explanted graft indicates that the material employed in the PEARS device is integrated into the peripheral adventitial tissue of the vessels without triggering surrounding inflammation or medial necrosis. Nevertheless, there was a uniform reduction in media thickness and smooth muscle cell atrophy following insertion into a PEARS device [182,183]. These preliminary alterations are troubling and indicate that the exostent impedes wall motion or mechanotransduction, commencing with the endothelial cell layer. Additional investigation is necessary in a true Ross procedure model to ascertain the influence on the mechanical characteristics of the neoaortic root.

The primary concern is the incremental dilation of the native ascending aorta. Additionally, the PA may undergo dilation at the site of the sinotubular junction, which can result in PA failure. To mitigate this risk, some recommend proactive management of the ascending aorta in patients with an ascending aortic diameter greater than 38–40 mm at the time of surgery. To stabilize the sinotubular junction, a short Dacron graft may be placed between the autograft and the ascending aorta. This prevents any impact on sinotubular junction diameters in the event of an increase in ascending aortic diameter [184,185,186]. In this instance, the detrimental effects on the elastic component of the vascular structure may be less severe when only a small part of the neoaorta is covered by the Dacron. It is crucial to acknowledge that the hinge function of the sinotubualar junction may be compromised due to the compressive influence of the outer Dacron [186,187,188,189,190,191,192].

**Figure 8:**
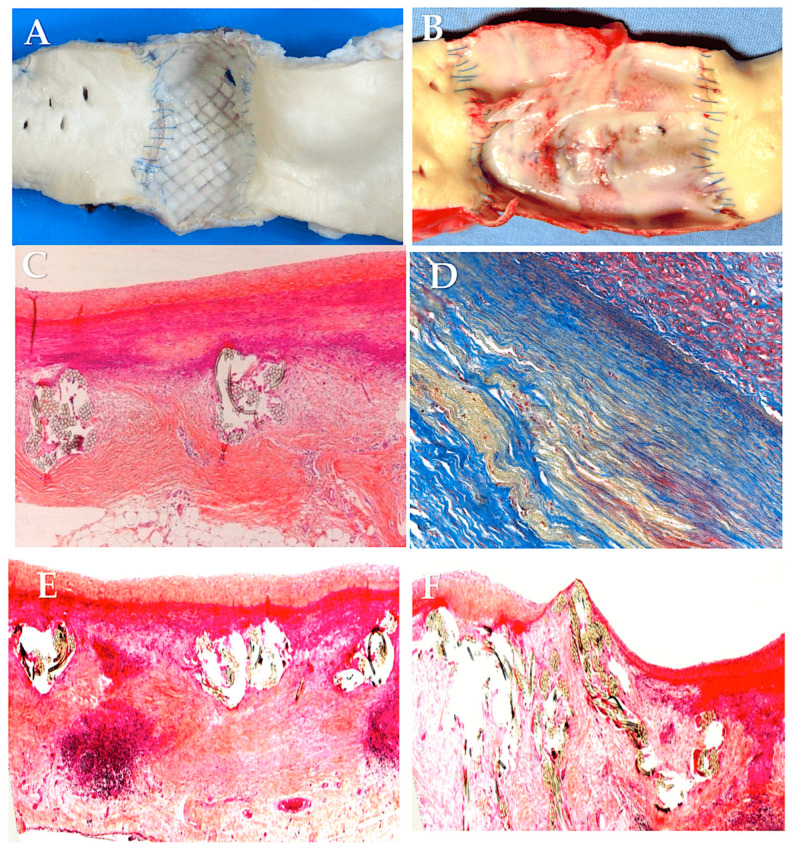
(**A**–**F**). Macroscopic and microscopic findings of external reinforcement of PA with non-resorbable polyester. (**A**) Mesh endoluminal transmigration of polypropylene. (**B**) Mesh endoluminal transmigration of polyethylene terephthalate. (**C**) Intimal hyperplasia and media with normal thickness and no disruption. (**D**) Muscular hyperplasia with fiber disorganization. (**E**,**F**) Endoluminal migration of the mesh.

The use of expanded polytetrafluoroethylene (ePTFE, Gore-Tex, WL Gore & Associates, Newark, Del.) as external reinforcement of the pulmonary autograft was investigated in a growing ovine animal model in two forms: as a non-resorbable mesh and as part of a semi-absorbable scaffold in conjunction with polydioxanone (PDF) (Figure 6). No instances of neoaortic root dilatation were observed in the absence of antihypertensive drug treatment [85,86,87,186,187,188,189,190,191,192]. The remodeling capacity of the pulmonary artery has been enhanced, while the negative effects of systemic pressure on the vessel wall have been mitigated. The aforementioned outcomes were accomplished through the utilization of a semi-bioresorbable vascular scaffold, comprising a combination of polydioxanone and expanded polytetrafluoroethylene [85,86,87,186,187,188,189,190,191,192]. In accordance with the extant literature and the findings of our experimental model of the Ross operation, the incorporation of a nonresorbable polyester reinforcement (Figure 5) can exert a considerable influence on the viability of tissue as a consequence of both the inflammatory response associated with the foreign body phenomenon and the biomechanical attributes of the reinforced pulmonary autograft [176,177,190,191,192,193,194,195,196]. The results of our investigation substantiate the occurrence of both macroscopic and microscopic alterations within the explanted graft. The histochemical analysis demonstrated the persistence of the nonresorbable polyethylene terephthalate within the PA wall, exhibiting evidence of partial migration and muscular hyperplasia with fiber disorganization (Figure 4) [85,86,87,187,197] (Figure 8).

**Figure 9:**
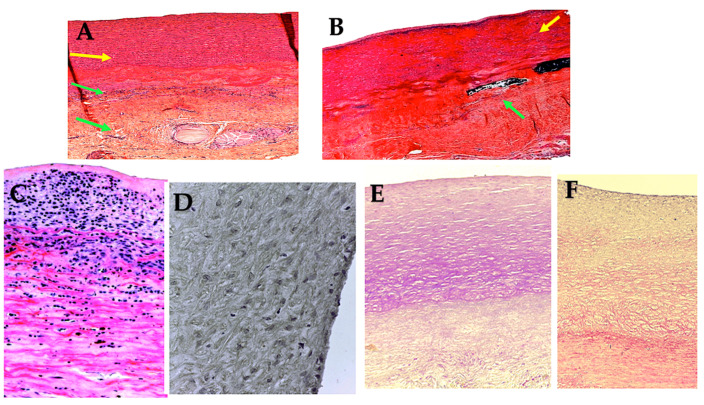
Histologic elastic remodeling of pulmonary autografts with resorbable polyester (polyglactin or polydioxanone) showed preservation of the endothelial lining and reorganization of the tunica media. Immediately beneath the intima, smooth muscle cells were found to be intertwined with collagen fibers. More deeply, the collagen bundles were intertwined with elastic fibers, forming a thick and highly organized layer of concentric lamellae. Loose connective tissue with adipocytes formed the tunica adventitia. (**A**) Intimal hyperplasia (yellow arrow) and media with normal thickness and no disruption (green arrow) are seen with the use of polyglactin mesh + polydioxanone. (**B**,**C**) The use of polydioxanone shows intimal hyperplasia (**B**) and media showing normal thickness and no disruption (yellow arrow) (**C**). Note the presence of PDS remnants (green arrow) (**D**–**F**). The use of polydioxanone as a reinforced external support favors the overexpression of metalloproteinase-9 in the reinforced group. This suggests an active process of extracellular matrix remodeling (**D**). (**E**) Mallory’s staining analysis shows an increase in the content of elastin fibers (pink). (**F**) Picrosirius red staining shows the formation of a compact collagen organization in the “elastic zone” of the vessel. The cellular infiltrate is less pronounced [86,87].

The findings of our investigations [1,34,35,85,86,87,187,188,189,190,191,192] indicate that the interaction between the provisional bioresorbable reinforcement and pulmonary autograft resulted in a sophisticated vascular remodeling process, which can be described as a dynamic equilibrium between inflammatory processes and extracellular matrix generation. This led to the formation of a “neovessel”, exhibiting characteristics comparable to native aortic vessels yet maintaining its biological activity and potential for growth following the resorption of the biomaterial. It was observed that the utilization of resorbable polyester (Polydioxanone or Polyglactin) resulted in augmented production of novel extracellular matrix. The matrix was distinguished by a greater concentration of elastin fibers within the PA and a more compact configuration of collagen fibers within the elastic zone of the vessel. These observations offer a reliable biological and biomechanical explanation for the reported improvement in clinical outcomes. It can thus be concluded that a biocompatible reinforcement of the PA would allow for the in vivo creation of a PA with morphostructural characteristics that improve tolerance to the hemodynamic load of the arterial system and ensure a harmonious increase in size during somatic growth [1,34,35,85,86,87,187,188,189,190,191,192] (Figure 9).

## 6. Future Perspectives

**Figure 10:**
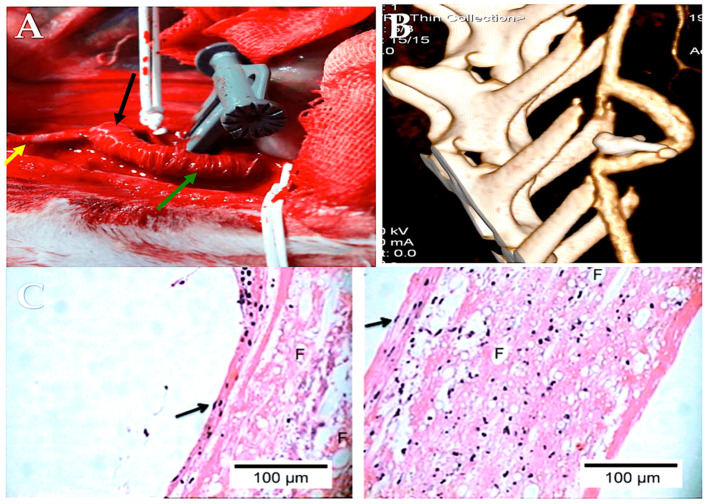
A TEVG was implanted in the abdominal aorta. The illustration depicts PLLA (Sigma-Aldrich) electrospun tubular scaffold that has been functionalized with heparin and utilized as a substitute for the abdominal aorta in a rabbit model. Panel (**A**) depicts an intraoperative photograph of the PLLA-armored scaffold that has been implanted and the ligature of the infrarenal aorta that has been placed between the two anastomoses. Panel (**B**) presents a three-dimensional reconstruction of the scaffold, created using maximum intensity projection and volume rendering algorithms. Panel (**C**): Histological analysis. The tissue was then subjected to a hematoxylin and eosin staining procedure. The scaffold exhibited a high degree of cellular colonization, with distinct phenotypic characteristics observed in different regions of the TEVG. The image on the left is a 40× magnification of the inner side of the TEVG. It is noteworthy that the flat, elongated cells with a protruding nucleus in the lumen (arrow) are organized in an endothelial-like fashion. The image on the right is a 40× magnification of the outer side of the TEVG. It is noteworthy that spindle-shaped cells, which are indicative of fibroblasts, can be observed with certainty (see arrow). The symbol F indicates the presence of polymer fibers in both the cross-sectional and longitudinal sections. Abbreviations: PLLA, poly-L-lactide; TEVG, tissue-engineered vascular graft [198].

Improved understanding of the predictors of pulmonary autograft dilatation and the underlying pathophysiology has focused attention on preventing this late complication. The provision of external support to the pulmonary autograft represents a logical solution to this problem. A number of studies have demonstrated that this approach can effectively prevent dilatation. Nevertheless, it is possible that this approach may also have an adverse effect on aortic root hemodynamics, which could theoretically negate the perceived improvements associated with the surgical procedure. To ascertain the actual impact of this approach, further research employing advanced imaging techniques is necessary. Additionally, bioengineered resorbable and semiresorbable scaffolds represent a promising avenue of investigation, with the potential to become a significant focus in future scientific inquiry within this domain. Such scaffolds could offer a promising solution by combining the benefits of an external support structure with the highly efficient hemodynamics observed in the unrestricted root (Figure 10).

The autograft’s morphological and geometric characteristics, as well as the heterogeneous elastic properties and the evolving mechanical attributes and strain patterns anticipated throughout the tissue’s growth and remodeling processes, whether reinforced or not, give rise to complex dynamics. Notwithstanding, certain ad hoc simplified schemes can be implemented to determine the key factors that predominantly influence the biomechanical response of reinforced and non-reinforced PAs subjected to systemic pressure. These schemes are governed by suitable geometric and mechanical parameters and may be considered as an alternative to complex, comprehensive models. Specifically, by invoking the comprehensive mechanical characteristics and mean initial autograft diameter d and wall thickness t of an equivalent elastic–cylindrical tube (e.g., the seminal work on growth and elasticity of arterial walls by Holzapfel and Ogden) [86,188,199] under the hypothesis of neo-Hookean incompressible hyperelasticity and leveraging the Laplace formula, one can mathematically derive the following relationship between the internal pressure *p* and the final deformed diameter *D* through the circumferential stress *s*:

$$p=s\times \frac{2T}{D}\Rightarrow p=G\times \left[1-{\left(\frac{d}{D}\right)}^{4}\right]\times \frac{2t}{d}$$

The aortic root anatomy is characterized by a heightened degree of intricacy, rendering it unsuitable for approximation to a cylindrical geometry. As previously outlined, material deformation occurs in both axial and lateral directions, and a shear stress modulus is employed to ascertain the sliding of conduit components. In light of these considerations, the PA reinforcement strategy must be evaluated in accordance with the mathematical model that has been developed and the initial experience that has been gained with resorbable reinforcement. The primary drawbacks associated with the utilization of synthetic materials in cardiovascular surgery pertain to their inability to adapt to the structural and growth dynamics of the vessels, as well as their capacity to elicit a robust inflammatory response that compromises the viability of autografts. This, in turn, disrupts the normal process of arterialization and impairs the elastic compliance of the grafts [1,200].

In this context, the objective is to identify a suitable material that meets the requisite shear modulus criteria while also demonstrating the desired differential dilation tendency in the root. From an elastomechanical perspective, ePFTE exhibits auxetic behavior, a unique property characterized by negative Poisson’s ratio. ePTFE possesses a negative Poisson’s ratio, which is a measure of a material’s capacity to react to external forces. This property endows ePTFE with highly advantageous compliance, as evidenced by its ability to undergo structural expansion in the direction opposite to tensile stress and, conversely, to contract in the direction of compression. Both PDF and poly-L-lactic acid are resorbable materials that can adapt their physicochemical properties to the elastomechanical behavior of PA, thereby promoting a physical remodeling process and playing a key role in the architecture of our proposed prosthetic system [86,188].

The semiresorbable crosslinked prostheses function on two different levels. In the initial stages, the mechanical properties of the material serve to provide elastic stiffness to the trunk, which helps to limit excessive vascular dilation and prevent aneurysm formation. Subsequently, as the material degrades, it gradually relinquishes its initial stiffening role, allowing pressure-induced PA tissue remodeling and growth. This ultimately leads to the arterialization of the vessel, and the overall outcome depends on the interaction between the material (PDF or poly-L-lactic acid) and the ePTFE in the semiresorbable crosslinked prostheses. From a biomechanical point of view, it is possible to imagine a virtuous cooperation between biological and synthetic materials, a kind of “stress-shielding” phenomenon that guides the physiological arterialization of the vessel wall, which ultimately determines the success of the pulmonary autograft system [86,188].

The advancement of wholly resorbable bioengineered scaffolds may facilitate sufficient time for pulmonary autografts to adapt to systemic pressure while also significantly reducing the risks inherent to the utilization of prosthetic materials. Furthermore, it would be prudent to conduct research on the medical treatment of patients who have undergone a Ross procedure. It may be possible to forestall PA expansion dilatation by more effectively regulating the risk variables. The elucidation of biomolecular pathways involved in ECM-mediated pulmonary autograft dilatation may facilitate the exploration of novel therapeutic avenues. In essence, the optimal solution to the issue of pulmonary autograft dilatation hinges on an integrated approach that encompasses a combination of personalized therapies and methodologies.

## 7. Conclusions

One of the most feared complications of the Ross procedure is pulmonary autograft dilatation. It is associated with the occurrence of aortic regurgitation and the requirement to reoperate. In order to maximize the benefits associated with the Ross procedure, efforts to minimize the risks of pulmonary autograft dilatation should be actively maintained. External support of the autograft effectively prevents dilatation. However, it may also interfere with the hemodynamics of the aortic root and have a detrimental effect on coronary perfusion. The risk of endocarditis may also be a potential issue with the use of prosthetic materials. A promising approach that integrates the advantages of early external support with the long-term benefits of normal root hemodynamics is temporary external support using bioengineered materials. To determine the long-term outcomes of these techniques and to further develop treatments to mitigate the risks of pulmonary autograft dilatation, additional clinical, bioengineering, and biomolecular research is needed in this area.

## Data Availability

Not applicable.
